# ^13^C labeling experiments at metabolic nonstationary conditions: An exploratory study

**DOI:** 10.1186/1471-2105-9-152

**Published:** 2008-03-18

**Authors:** Sebastian Aljoscha Wahl, Katharina Nöh, Wolfgang Wiechert

**Affiliations:** 1 Bioprocess Engineering, Max Planck Institute for Dynamics of Complex Technical Systems, Magdeburg, Germany; 2 Fermentation Technology, Institute of Biotechnology 2, Research Centre Jülich GmbH, Jülich, Germany; 3 Department of Simulation, Institute of Systems Engineering, University of Siegen, Siegen, Germany

## Abstract

**Background:**

Stimulus Response Experiments to unravel the regulatory properties of metabolic networks are becoming more and more popular. However, their ability to determine enzyme kinetic parameters has proven to be limited with the presently available data. In metabolic flux analysis, the use of ^13^C labeled substrates together with isotopomer modeling solved the problem of underdetermined networks and increased the accuracy of flux estimations significantly.

**Results:**

In this contribution, the idea of increasing the information content of the dynamic experiment by adding ^13^C labeling is analyzed. For this purpose a small example network is studied by simulation and statistical methods. Different scenarios regarding available measurements are analyzed and compared to a non-labeled reference experiment. Sensitivity analysis revealed a specific influence of the kinetic parameters on the labeling measurements. Statistical methods based on parameter sensitivities and different measurement models are applied to assess the information gain of the labeled stimulus response experiment.

**Conclusion:**

It was found that the use of a (specifically) labeled substrate will significantly increase the parameter estimation accuracy. An overall information gain of about a factor of six is observed for the example network. The information gain is achieved from the specific influence of the kinetic parameters towards the labeling measurements. This also leads to a significant decrease in correlation of the kinetic parameters compared to an experiment without ^13^C-labeled substrate.

## Background

The recent developments in Metabolomics and Fluxomics open up new perspectives for detailed *in vivo *studies of the cellular metabolism. Various experimental approaches using GC- or LC-MS enable the measurement of intracellular concentrations and isotope enrichments with increasing accuracy. Nowadays, almost all metabolites of the central carbon metabolism can be detected [1-3]; although not all can be quantified exactly. Metabolic fluxes depend on the metabolite concentrations, enzyme concentrations (regulated by transcription [4]) as well as regulatory mechanisms (like enzyme modification [5] and allosteric inhibition or activation [6]). Different information about the *in vivo *mechanisms and fluxes can be collected from already established experimental approaches.

**Figure 1:**
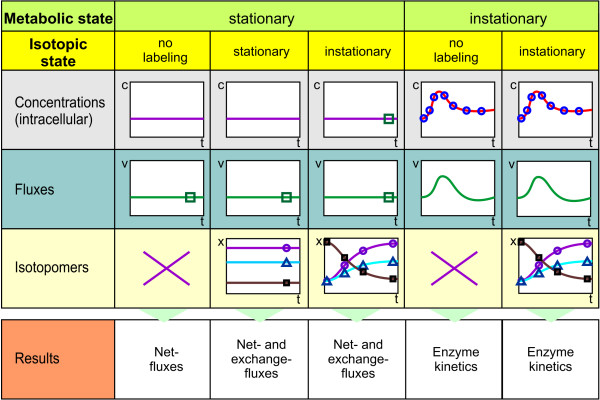
**Category of experiments**. Overview of experiments used for the estimation of intracellular fluxes and enzyme kinetics. The new metabolic and isotopic nonstationary state is analyzed here within a simulation study.

Figure 1 depicts the most prominent approaches in Fluxomics and Metabolomics and their information targets. In general, experimental approaches can be divided into metabolic stationary and metabolic nonstationary experiments. Whereas metabolic stationary approaches aim to quantify intracellular fluxes, nonstationary experiments are used to identify reaction kinetic properties like substrate affinities and allosteric inhibition. Fluxes at different metabolic states can only be calculated from a reaction kinetic model (with identified parameters). The following approaches are currently established:

1. Metabolic Flux Analysis (left state in Figure 1): At metabolic steady state it is assumed, that the intracellular concentrations and fluxes are not changing during the time of the analysis. A model based on mass balances is used to quantify the flux through the network [7].

2. ^13^C Metabolic Flux Analysis (second left state in Figure 1): It is well known that solely stoichiometric balancing will not lead to a resolution of bidirectional or parallel fluxes [8]. Experiments with additional ^13^C labeled substrates have been shown to increase the measurable information and to enable the quantification of exchange fluxes and parallel fluxes [9,10].

3. ^13^C Metabolic Flux Analysis at isotopically nonstationary state (third state in Figure 1). Here the organism is kept at metabolic stationary state while a ^13^C labeled substrate pulse is applied. Only recently, Nöh et al. [11] have evaluated an experiment of this type. It was shown that the duration of the labeling experiment can be drastically reduced from several hours to the order of minutes.

4. Stimulus-Response Experiments (fourth state in Figure 1). At a metabolic nonstationary state the intracellular concentrations (and consequently also the fluxes) are not constant. These conditions target the identification of *in vivo *enzyme kinetics with one single experiment. The currently most prominent approach in this field is given by Stimulus-Response Experiments [12-15]. A culture is driven to a substrate limited state and then excited by a strong external stimulus, typically a substrate pulse. The metabolic response of the cells is tracked by rapid sampling on a sub-second timescale [16-18]. Although lots of data is generated from a stimulus response experiment [16,19], the approach has shown to be limited: still only a part of the parameters can be identified. Even if the data from several experiments is gathered, it is not possible to determine all kinetic parameters [20].

These experiments are now common procedure. Yet missing is the experiment that results from a fusion of approach 3 and 4, i.e. labeling the substrate in a metabolically dynamic experiment. This type of experiment is theoretically analyzed to evaluate whether this type of labeling experiment can increase the parameter estimation accuracy of a reaction kinetic model. To this end, it is first shown how the experiments can be modeled based on the known concepts from ^13^C Flux Analysis and reaction kinetic modeling. A series of papers [21-23] are concerned with the integration of kinetic and isotopic information. In contrast to the present integration, the authors assumed most enzyme kinetic parameters to be known. Moreover the system was analyzed in a metabolic steady state and therefore resembles approaches 2 and 4 under the perquisite of mostly known kinetic parameters. The values for these parameters are taken from literature or additional experiments [23]. The approach presented here is based on a dynamic experiment rather than one or several metabolic steady states and all enzyme kinetic parameters are to be estimated from the experimental data.

The approach is based on a complete isotopomer model that is capable of exploiting all available isotopomer measurements from MS(/MS) or NMR devices. In order to demonstrate the general mathematical framework a small example reaction network is discussed the first time. Comparing the outcomes of an experiment with labeling to those of a reference without the addition of ^13^C substrate the information surplus from the metabolic and isotopic transient state is elucidated. To cover different experimental settings, three scenarios of available measurements are introduced and compared.

### Modeling metabolic and isotopic nonstationary systems

A general model structure for metabolic and isotopic nonstationary systems can be obtained by combining models from both worlds. However, this hybrid structure also inherits some standard assumptions from kinetic and isotopic models. Precisely, all upcoming equations are only valid if the following assumptions [24] are accepted:

1. Homogeneity: All intracellular metabolites are distributed uniformly.

2. No isotopic effects: The enzymatic reactions are not influenced by the labeling state of the metabolites.

3. Fast equilibrium of enzyme complexes: Intermediate enzyme complexes rapidly reach equilibrium such that the quasi-stationary assumption of Michaelis-Menten [6] is valid.

### Reaction kinetic model

With these assumptions, well known mechanistic or approximate enzymatic rate laws like Michaelis-Menten [6] can be used to describe the fluxes **v **in dependence of substrate, product and effector concentrations, kinetic parameters **α **(like *v*_*max*_, *K*_*M*_) and external (experimental) parameters **β **(like substrate feed):

(1) **v **= **v**(**c**, **α**, **β**)

The concentrations of metabolites (**c**) do change depending on the in- and effluxes **v**. The mass balances of the pools can be formulated using the well known stoichiometric matrix **N **[25]:

(2) $$\dot{c}=N\cdot v\left(c,\alpha ,\beta \right)$$

### ^13^C Balance equations

Here, the labeling state is modeled using the isotopomer concept [24] to keep the contribution understandable on a basic level. Nevertheless, more evolved but mathematically equivalent concepts like cumomers [26], bondomers [27] or elementary metabolite units [28] can also be used. A metabolite with *n *carbon atoms has 2^*n *^labeling states. Thus, 2^*n *^isotopomer fractions are required for each metabolite to describe its labeling state. The overall isotopic state of the network is described by a vector **x **containing the isotopomer fractions of all metabolites. Isotopomer dynamics of a metabolite pool depends on the pool concentration **c**, the input labeling **x**^inp ^and the flux functions **v**. The overall system for the labeling state is given by [29]:

(3) $$⍂\left(c\right)\cdot \dot{x}=f\left(v\left(c,\alpha ,\beta \right),x^{\text{inp}},x\right)$$

Here, the operator ⍂(c) generates a diagonal matrix composed of concentrations **c**_*i *_(with *n*_*i *_repetitions which correspond to all isotopomer fractions, *n*_*i *_being the number of isotopomers of metabolite *i*). The nonlinear function **f **includes the stoichiometry of the isotopomer transitions, the vector **x **describes the isotopomer distribution of the balanced intracellular pools (for an example take a look at Eq. (14) in the example section) and **x**^inp ^is defined by the isotopomer fractions of the input substrate.

Notice that unlike in stationary isotopomer balances [30], the vector **v **is not constant but changes over time in dependence of the metabolite concentrations. Combining both model equations (2) and (3), the metabolic and isotopic transients can now be completely described. Hence, the resulting model is a hybrid of a classical ^13^C labeling and a reaction kinetic model. The functions **f **is constructed based on matrix calculus as described earlier by Wiechert et al [24] but replacing the vector **v **with a vector of kinetic rate terms. A clear difference appears at the point of solution; Instead of a numeric equation solver, numeric integration is applied to calculate the labeling transients.

### Measurement model

The measurement model is a function that couples the dynamic isotopomer model with the available measurements. Within this section the measurement model will be described only very briefly in order to avoid rather unnecessary technical explanations (these can be found in Appendix A). Generally, at time point *t*_*k *_measurements of the labeling and the concentrations are expected. Both quantities are gathered in the measurement vector **y**(*t*_*k*_) that depends on the state of the system **c**(*t*_*k*_) and **x**(*t*_*k*_). Hence, we introduce a measurement function **g**:

(4) **y**(*t*_*k*_) = **g**(**x**(*t*_*k*_), **c**(*t*_*k*_)), *k *= 1,...,*N*

Collecting all sampling time points into a vector **ξ **= (*t*_1_,...*t*_*N*_)^*T*^, Eq. (4) compactly reads:

(5) $$\underset{y\left(\xi \right)}{\underset{︸}{\left(\begin{array}{c} y\left(t_{1}\right)\\ y\left(t_{2}\right)\\ \vdots \\ y\left(t_{N}\right) \end{array}\right)}}=\underset{g\left(x\left(\xi \right),c\left(\xi \right)\right)}{\underset{︸}{\left(\begin{array}{c} g\left(x\left(t_{1}\right),c\left(t_{1}\right)\right)\\ g\left(x\left(t_{2}\right),c\left(t_{2}\right)\right)\\ \vdots \\ g\left(x\left(t_{N}\right),c\left(t_{N}\right)\right) \end{array}\right)}}$$

A concrete function **g **is presented within the example given in the appendix for massisotopomer measurements.

### Parameter sensitivity

The final goal of the nonstationary experiment is the determination of the kinetic parameters **α**. All other quantities (fluxes **v**, concentrations **c**) follow from these parameters. Sensitivities quantify the influence of **α **on the model response, namely the concentration time course **c**(t) and the isotopomer distributions **x**(t). Implicit derivation of the differential equations (2) and (3) with respect to the parameters **α **leads to the sensitivity differential equations associated with the model (2)–(3):

(6) $$\begin{array}{c} \overset{•}{\frac{dc}{d\alpha }}=N\cdot \left[\frac{dv}{d\alpha }+\frac{dv}{dc}\cdot \frac{dc}{d\alpha }\right]\\ \;⍂\left(c\right)\cdot \overset{•}{\frac{dx}{d\alpha }}=\frac{df}{dv}\cdot \frac{dv}{d\alpha }+\frac{df}{dx}\cdot \frac{dx}{d\alpha }-\frac{d□\setminus }{dc}\cdot \frac{dc}{d\alpha }\cdot \dot{x} \end{array}$$

Besides the influence of the parameters **α **on the system state, the influence on the expected measurements is now easily calculated using the chain rule:

(7) $$\frac{dy}{d\alpha }=\frac{dg}{dx}\cdot \frac{dx}{d\alpha }+\frac{dg}{dc}\cdot \frac{dc}{d\alpha }$$

As shown in the following sections, the sensitivities are essential for the statistical evaluation and comparison of different experimental configurations [10].

### Nonlinear regression model

Regression analysis is the chosen method to estimate the kinetic parameters from the experimental data. Assuming that the model adequately describes reality and that experimental measurements **y **have some unknown error **ε**, Eq. (5) extends to:

(8) **η**(**ξ**) = **g**(**x**(**ξ**), **c**(**ξ**)) + **ε**(**ξ**)

Parameter estimation is performed by using weighted least square minimization. Expecting that the errors **ε**(**ξ**) are normally distributed with expectation **E**[**ε**(**ξ**)] = **0 **and the given measurement covariances are collected in the measurement covariance matrix **Σ**, the weighted least squares functional for the calculation of the optimal parameters $\hat{\alpha }$ reads [31]:

(9) $$\hat{\alpha }=\arg\underset{\alpha }{\min}{\left(\eta -g\right)}^{T}\sum^{-1}\left(\eta -g\right)$$

Various algorithms can be applied to solve this minimization problem like derivation-free simplex methods [32], evolutionary strategies [33], or gradient-based methods [34].

### Statistical evaluation

In order to obtain a statement about whether or not the information contained in the measurements is sufficient to identify the parameter values within a certain precision, it is essential to analyze the accuracy of the parameter estimation $\hat{\alpha }$ found by solving Eq. (8). Assuming that the estimate $\hat{\alpha }$ is close to the real solution α* application of linearized regression analysis is feasible. In experimental design studies usually a parameter set $\hat{\alpha }$ = α* is chosen. It is known, that linear statistics can produce imprecise estimates of parameter confidence regions [35]. Nevertheless, this tool still fulfils its purpose when it is used to compare different experiments. Here the relative improvement of the confidence regions is much more important than their precise knowledge. Moreover, the concept of correlation coefficients that characterizes the confidence regions is an inherently linear concept. It will turn out that parameter correlations are relevant because with ^13^C labeled substrates parameter correlations strongly decrease that lead to smaller confidence regions.

The covariance of the parameters $\hat{\alpha }$ is calculated using the derivative and the covariance of the measurements **Σ **by [36]:

(10) $${\mathrm{cov}}^{-1}\left(\hat{\alpha }\right)={\left(\frac{dy}{d\alpha }\right)}^{T}\Sigma^{-1}\left(\frac{dy}{d\alpha }\right)$$

In the appendix section it is explained how the different types of measurements and additional knowledge are integrated.

### Statistical measures to compare experiments

The parameter covariance matrix $\hat{\alpha }$ contains all information needed to judge the information gain of an experiment, namely parameter standard deviations, correlations and confidence regions. A drawback of using the covariance matrix is its size. Even for small models it is rather difficult to evaluate and compare experiments on its basis. Therefore statistical measures are used that reduce the complexity and facilitate an automated comparison of experiments. Usually, a reference experiment is specified to which all other experiments are compared.

A commonly used measure is the *D*-criterion, which is calculated from the determinant of the covariance matrix. It comprehensively summarizes the available parameter information. To compare different experiments Möllney et al. [10] propose to use a root of the *D*-criterion. The scaled ratio *I*_*D *_between an experiment and the reference experiment (index *ref*) is defined by:

(11) $$\begin{array}{cc} I_{D}=\sqrt[{2\;\dim(\alpha )}]{\frac{D_{\text{ref}}}{D}}, & D=\det\left(\mathrm{cov}\left(\hat{\alpha }\right)\right) \end{array}$$

Roughly speaking the *I*_*D *_value reflects an overall information gain based on the volume of the covariance ellipsoid (for details see [10]). The standard deviations and correlations of the judged experiment are – on an average – *I*_*D *_times smaller than those from the reference experiment. However, this does not guarantee that every single standard deviation is improved.

Besides the *D*-criterion, the *A*-criterion is frequently applied. The latter criterion reflects the mean of the diagonal elements of the covariance matrix. Again, a scaled scalar is introduced to compare two experiments:

(12) $$\begin{array}{cc} I_{A}=\sqrt{\frac{A_{\text{ref}}}{A}} & A\left(\hat{\alpha }\right)=\frac{1}{N}\;\text{trace}\left(\mathrm{cov}\left(\hat{\alpha }\right)\right) \end{array}$$

In contrast to the *D*-criterium, correlations between parameters are not taken into account; the *A*-criterium is based on the parameter variances only. Therefore, the square root has to be taken.

### An exploratory study

Within this section the described framework is applied to an example network. As an example, some equations are explicitly shown to facilitate the understanding of the presented concepts. Some different conceivable scenarios regarding the available measurements are also introduced to cover different measurement setups and analyze the influence of, for example, a missing concentration measurement.

### Example network

**Figure 2:**
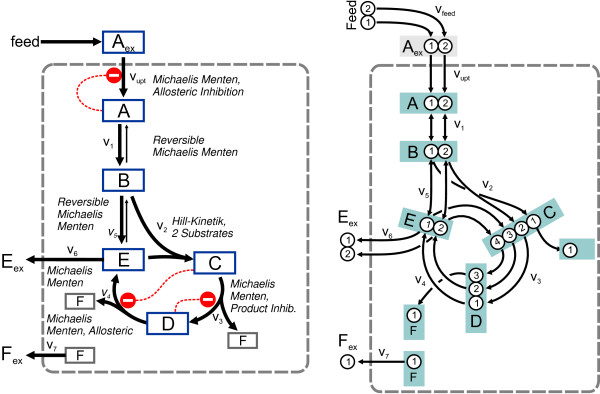
**Example network structure including atom transitions**. The reaction kinetics are given in Table 1.

The network chosen for this study is shown in Figure 2. Although it is simple, it nonetheless reflects all typical reaction mechanisms of the central carbon metabolism. A linear reaction sequence (v_upt_, v_1_, v_2_) with a feedback inhibited entry reaction (v_upt_) as well as a cyclic reaction sequence (v_3_, v_4_) with some fillup reaction (v_5_) and an exit (v_6_) is included. Some reversible reaction steps (v_1_, v_5_) are incorporated as well as a bimolecular reaction step (v_2_) with Hill-type kinetic mechanism and irreversible split reactions (v_3_, v_4_) are present (see Table 1 for a complete listing of reaction mechanisms and the kinetic expressions).

**Table 1:** Kinetic rate laws used in the small example network.

Reaction	Mechanism	Inhibitor	Kinetic
upt	Michaelis-Menten, 1 Inhibitor	A	$\frac{v_{\max}A}{K_{mA}\left(1+\frac{A}{K_{I}}\right)+A_{ex}\left(\frac{A}{K_{I}}\right)}$
v1	reversible Michaelis-Menten		$\frac{v_{\max}\left(A-\frac{B}{K_{eq}}\right)}{K_{mA}\left(1+\frac{B}{K_{mP}}\right)+A}$
v2	2 Substrate Hill-Kinetic		$\frac{v_{\max}B^{hA}E^{hB}}{\left(K_{mA}+B^{hA}\right)\left(K_{mB}+E^{hB}\right)}$
v3	Michaelis-Menten, 1 competitive Inhibitor	D	$\frac{v_{\max}C}{K_{mA}\left(1+\frac{D}{K_{I}}\right)+C}$
v4	Michaelis-Menten, 1 competitive Inhibitor	C	$\frac{v_{\max}D}{K_{mA}\left(1+\frac{C}{K_{I}}\right)+D}$
v5	reversible Michaelis-Menten		$\frac{v_{\max}\left(A-\frac{E}{K_{eq}}\right)}{K_{mA}\left(1+\frac{E}{K_{mP}}\right)+B}$
v6	Michaelis-Menten		$\frac{v_{\max}E}{K_{mA}+E}$

The assumed metabolic regulation reflects some basic principles like product inhibition (reaction v_upt _by A, v_2 _by C) and allosteric inhibition (v_4 _by C). Short chain molecules were chosen to keep the dimension of the isotopomer equations low. The substrate of the system contains only 2 carbon atoms (A_feed_) and the molecule formed in the bimolecular reaction entering the cycle has 4 carbon atoms.

### Isotopomer balance equations and simulation

Some assumptions about the experimental setup and the network will be needed to perform the simulation study of the nonstationary state: (1) the external influences **β**, (2) assumptions about the reaction mechanisms that will form the functions **v **and (3) estimates for the unknown kinetic parameters **α**.

**Figure 3:**
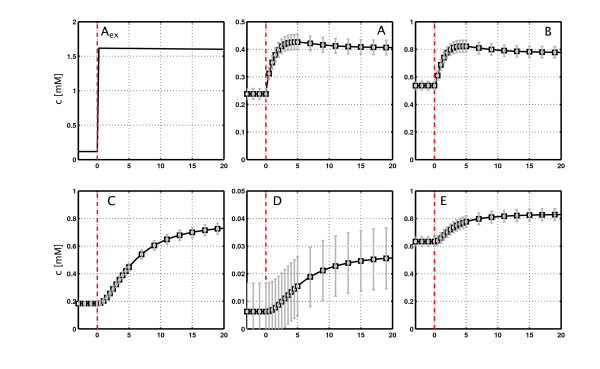
**Simulation of the example network and the assumed measurement standard deviations**. The extracellular concentration (A_ex_) rises from 0.05 mM to 1.5 mM by the pulse. The intracellular concentrations A and B show a short overshoot, C, D and E increase continuous and reach the steady state after about 25 s. The squares show the noise free measurements generated from the simulation. The standard deviations include the errors from sample processing and the MS measurements.

The balance equations for the metabolite pools are not shown here because they are well documented in literature [7]; five pools are balanced for the example network. In total, 24 kinetic parameters are needed for the reaction kinetics. In order to model the reactor feeding profile, one input parameter (${\dot{v}}_{\text{feed}}$) is introduced. The values used within this study are listed in Table 2. Having the balances in hand, the time course of the concentrations **c**(*t*) is calculated by integrating Eq. (2). Figure 3 shows the simulation result. Concentrations of the metabolite pools A and B show a short overshoot, while concentrations C, D and E show a monotonic increase after the pulse. Pools A and B rapidly reach a steady state after about 10 s, while the dynamics in C, D and E do not reach a new steady state in the given simulation time of 20 s.

**Table 2:** Kinetic parameters for the simulation study.

Reaction	Parameter	Value
feed	*c*	0.01
upt	ν_max_	1
	*K*_*I*_	2
	*K*_*mA*_	0.001
v1	ν_max_	3
	*K*_*eq*_	3
	*K*_*mA*_	0.1
	*K*_*mP*_	3
v2	ν_max_	2.5
	*K*_*mA*_	0.25
	*hA*	2
	*K*_*mB*_	2
	*hB*	3
v3	ν_max_	2
	*K*_*mA*_	2
	*K*_*I*_	0.05
v4	ν_max_	3
	*K*_*mA*_	0.1
	*K*_*I*_	1
v5	ν_max_	2
	*K*_*eq*_	4
	*K*_*mA*_	1
	*K*_*mP*_	1
v6	ν_max_	2
	*K*_*mA*_	3

The atom transitions have to be known to balance the isotopomer fractions (Eq. (3)). These are shown in Figure 2 and are also listed in Table 3. Forty isotopomer fractions are balanced in the example. Four input variables are needed to describe the labeling of A_ex _(here set to **x**^inp ^= (0.01,0.01,0.01,0.97)^T^). Exemplary, the balance equations for the isotopomer fractions **a **= (a_00_, a_01_, a_10_, a_11_)^T ^of pool A are shown.

**Table 3:** Atom transitions for the small example network

Flux	Substrates				Products		
v_feed_	Feed			>	A_*ex*_		
	#ab				#ab		
v_upt_	A_ex_			>	A		
	#ab				#ab		
v_1_	A			>	B		
	#ab				#ab		
v_2_	B	+	E	>	C		
	#ab		#cd		#abcd		
v_3_	C			>	D	+	F
	#abcd				#bcd		#a
v_4_	D			>	E	+	F
	#ab				#ab		#c
v_5_	B			>	E		
	#abc				#ab		
v_6,ex_	E			>	E_ex_		
	#ab				#ab		
v_7,ex_	F			>	F_ex_		
	#a				#a		

The notation of Wiechert and de Graaf [24] is used.

(13) $$\begin{array}{c} \frac{dA}{dt}=v_{upt}^{\to }-v_{1}^{\to }+v_{1}^{\leftarrow }\\ A(t)\frac{da_{00}}{dt}=a_{ex00}\;v_{upt}^{\to }-a_{00}\;v_{1}^{\to }+b_{00}\;v_{1}^{\leftarrow }\\ A(t)\frac{da_{01}}{dt}=a_{ex01}\;v_{upt}^{\to }-a_{01}\;v_{1}^{\to }+b_{01}\;v_{1}^{\leftarrow }\\ A(t)\frac{da_{10}}{dt}=a_{ex10}\;v_{upt}^{\to }-a_{10}\;v_{1}^{\to }+b_{10}\;v_{1}^{\leftarrow }\\ A(t)\frac{da_{11}}{dt}=a_{ex11}\;v_{upt}^{\to }-a_{11}\;v_{1}^{\to }+b_{11}\;v_{1}^{\leftarrow } \end{array}$$

Again it must be noted that the fluxes are functions of kinetic parameters (see Table 1). For example, the flux v_1 _(A → B) is a reversible flux. Its rate is given by:

(14) $$v_{1}=\frac{v_{\max}\left(A-\frac{B}{K_{eq}}\right)}{K_{mA}\left(1+\frac{B}{K_{mP}}\right)+A}$$

**Figure 4:**
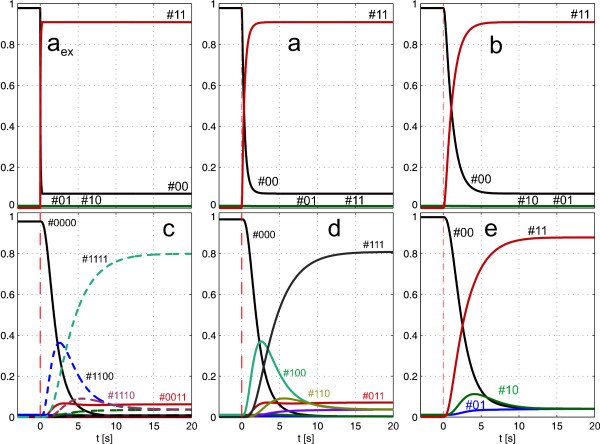
**Simulation of the isotopomer enrichments**. The extracellular substrate A_ex _immediately switches from unlabeled to labeled. The enrichment of A and B is fast (steady state after <10 s), pool C shows intermediate labeling patterns before reaching the fully labeled steady state. These intermediate patterns can also be seen in D and E.

With known initial isotopomer fractions **x**_0 _at time point *t *= 0 (usually determined by the natural isotope enrichments), the integration of Eq. (3) yields the time course **x**(*t*) of the isotopomer enrichments. In Figure 4 it can be seen that pools A and B are rapidly enriched with labeled carbon. Only at 2 s respectively at 5 s after the pulse they do show the labeling of the input substrate A_ex_. Pools C, D and E exhibit a different behavior. Especially in C and D an interim labeling state can be observed, that results from the reaction of unlabeled E#00 with fully labeled B#11. This state is still seen in D while in E it is not as pronounced because E is fed from B and D. This already hints at additional information contents, because a more detailed behavior in comparison to the one shown Figure 3 is observed. In the following sections, it will be seen that the kinetic parameters do influence the isotopomer distribution differently to the concentration time course.

### Scenarios

**Figure 5:**
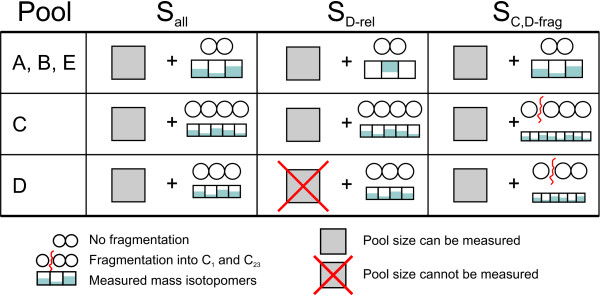
**Summary of the compared measurement scenarios**. In S_all _it is assumed, that all concentrations and mass isotopomers can be measured. Scenario S_D-rel _is less informative, the concentration of metabolite D cannot be quantified. S_C,D-frag _assumes MS/MS measurements with fragmentation for the metabolites C and D.

Since there is still much development in the analytical methods, scenarios with different measurements are investigated. In order to estimate the influence of increased or decreased availability of measurement information the following cases will be compared (summarized in Figure 5):

• Scenario S_all_: This is an optimistic scenario where all metabolite concentrations can be quantified and all mass isotopomer fractions are available. Here, the mass isotopomers of the entire carbon skeleton are measured without any fragmentation.

• Scenario S_D-rel_: Here, less information is available compared to S_all_; this scenario is derived from findings that for some metabolites the concentration could not be determined [11]. Nevertheless, it was possible to measure the mass isotopomer ratios. As an example, the concentration of D cannot be determined, but its mass isotopomer ratios are measured.

• Scenario S_C,D-frag_: Compared to scenario S_all _additional mass measurements of fragments are available. As an example, the labeling distribution of the pools C and D are measured in more detail because of a specific fragmentation in MS/MS measurement mode (see Figure 5). Metabolite D fragments into a C_2 _and a C_1 _skeleton. Here, the mass isotopomers of the C_2 _body can be measured additionally. Instead of four mass isotopomers, in total six tandem mass isotopomer measurements are generated [37]. Metabolite C fragments into a C_3 _and a C_1 _body. Consequently, instead of five mass isotopomer ratios, eight tandem mass isotopomers are measured.

To summarize, scenario S_all _is optimistic, S_C,D-frag _is even more optimistic, whereas in S_D-rel _some information is missing.

### Sampling and quality of the measurements

Besides the measurements, the sampling frequency is also crucial for parameter identification [29]. For all scenarios, the following sampling setup is used:

• Three samples are taken every 1 s before the pulse (-3...0 s),

• In the following 5 s samples are taken every 0.5 s (0.5...5 s),

• After 7 s samples are taken every 2 s (7...19 s)

**Figure 6:**
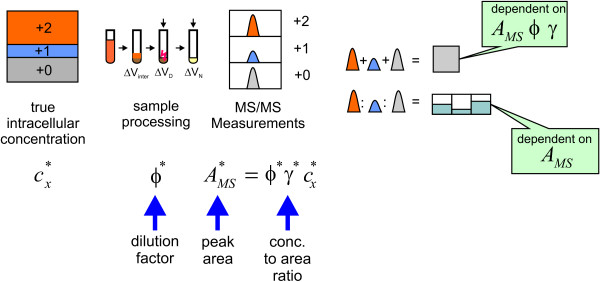
**Scheme of the sample processing steps**. The single sampling steps and their effects on concentration and labeling measurements.

The quality of the measurements depends on the accuracy of the measurement device and possible errors during sample preparation. Figure 6 depicts the sampling steps usually needed to determine intracellular concentrations. Essentially, the sampling steps lead to a dilution φ. The signal intensity depends on the concentration in the sample and the response factor **γ **that is determined from standard additions [38]. More details about the measurement model can be found in the appendix section. All sampling steps and possible errors have to be taken into account for the statistical analysis. Based on the current knowledge for the standard deviations the following factors are considered:

• The response factors **γ **(set to 1 if determined) have a standard deviation **σ**_γ _of 1%.

• The dilution factors **φ** (**ξ**) (also set to 1) have a standard deviation of 3%.

• The MS(/MS) measurement (area) itself is composed of a relative error which is assumed in the order of 1% and an absolute error that was set to 10 nM. The latter one appears rather high, but as the dilution usually will be around a factor 20 it is within a realistic range:

(15) σ_*MS*_(*t*_*k*_) = 10 nM + 0.01 γ *y*_*MS*_

## Results

### Statistical analysis

The example system consists of 40 isotopomer and six concentration states with 24 parameters. Hence, the parameter sensitivity matrix (Eq. (6)) contains 46·24 = 1104 entries for each of the 19 sampling time points. Although interesting, a detailed analysis of these large matrices is extraordinarily time-consuming. In fact, the influence on the measurable signals is more relevant. As it can be seen from equations, and the measurement equations are linear. Thus, the calculation of the output sensitivity is straight forward (Eq. (7)).

**Figure 7:**
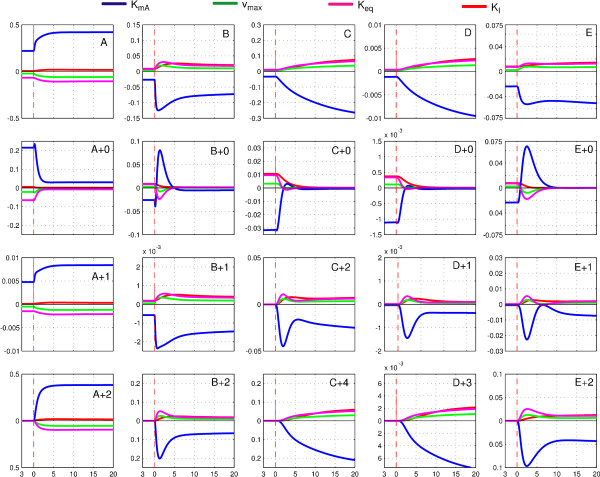
**Parameter sensitivity plots**. First row: Sensitivities of the concentrations to the parameters of reaction v_1 _(K_mA_, v_max _and K_eq_) and v_upt _(K_I_). Second to last row: Sensitivities of selected mass isotopomer concentrations to the same parameters.

Figure 7 shows the course of the sensitivities with respect to kinetic parameters of the uptake reaction v_upt _(*K*_*I*_) and reaction v_1 _(*K*_*mA*_, *v*_*max *_*and K*_*eq*_). The sensitivities have to be compared on a comparable scale. Recall that the mass isotopomers are given as fractions of the pool. Multiplying the fractions with the pool concentration, the mass isotopomer concentrations are immediately obtained by **c**·**x**.

These quantities can be directly compared to metabolite concentrations. Their parameter sensitivities can be simply obtained from Eq. (7) by using the chain rule. The sensitivity matrix d**y**/d**α **includes the sensitivities of the concentrations and of the mass isotopomers with respect to kinetic parameters. These sensitivities are plotted in Figure 7. It is observed that:

• After the pulse there is a strong increase in sensitivity of mass isotopomer concentrations of fully labeled pools (A+2, B+2, E+2, with a less extend C+4, D+3)

• The sensitivity of unlabeled concentrations (A+0, B+0, C+0, D+0, E+0) reaches zero, with some intermediate peaks.

• Some parameters show maxima in sensitivity, e.g. d(B·b_+0_)/dK_mA_, d(C·c_+2_)/dK_mA_, d(E·e_+2_)/dK_mA_.

• Some parameters first show positive, then negative influence on the mass isotopomers (and vice versa), e.g. d(B·b_+0_)/dK_mA_, d(B·b_+0_)/dK_eq_, d(B·b_+0_)/dv_max_.

• Most parameter sensitivities reach a new steady state within the given simulation interval of 20 s, e.g. d(A·a_+2_)/dK_mA_, d(A·a_+2_)/dK_eq_, d(A·a_+2_)/dv_max_.

These findings clearly show that the kinetic parameters do not only influence the total metabolite concentrations, but also influence the labeling dynamics. Within the statistical analysis section it will be seen that this will help to achieve an identification of the enzyme parameters.

### Parameter standard deviations

**Figure 8:**
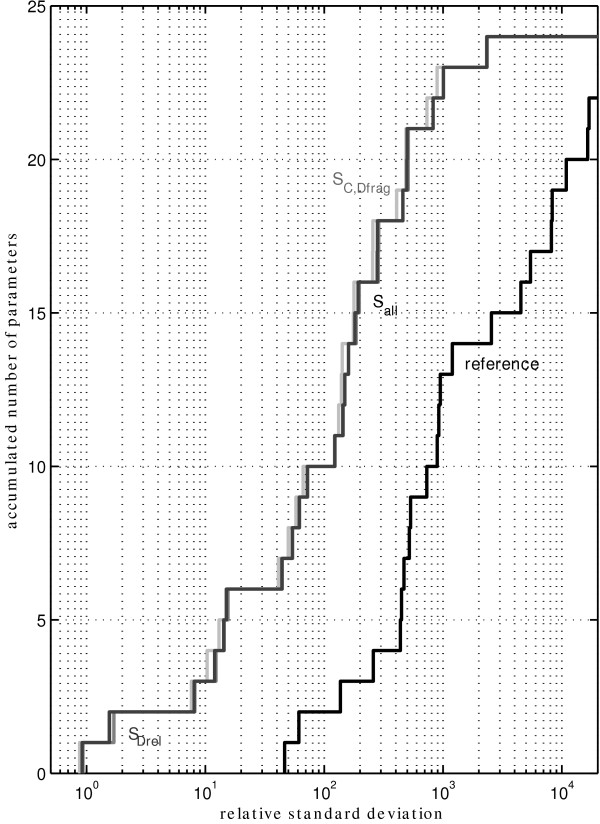
**Cumulative distribution of the estimated standard deviations**. In the unlabeled reference experiment the most accurate parameter is determined with a relative standard deviation of about 46% (first step of the black line). Only two parameters can be determined with less than 70% standard deviation. In contrast, using a labeled substrate, 10 parameters (about 40% of the parameters) can be determined with a standard deviation of less than 70%.

Figure 8 shows a cumulative plot of the estimated relative standard deviations of all parameters. Obviously, the majority of the parameters can hardly be determined from the (unlabeled) reference experiment. The best estimate is found to have about 46% relative error. The second step is seen at about 61%, which is the second best estimate. Three parameters are found within a standard deviation of about 137%. These high deviations are observed because the scenario includes 3% sample dilution error and additional noise from the MS device. Especially for low concentrated metabolites (like E, see Figure 3), high standard deviations occur due to the ground noise (10 nM) of the MS measurements. It has to be taken into account too, that there are more than the 24 kinetic parameters. Another 19 × 5 + 5 = 100 parameters (five φ 's for each measured metabolite and each sampling time point and five γ 's) are introduced for the measurement model. Although these parameters are measured directly from the sampling volume, added reagents and standard additions, these additional parameters do consume some information. It is seen from the statistical analysis that these parameters are also influenced and their estimation accuracy gets higher compared to the experimental errors.

A pronounced increase in estimation accuracy of the kinetic parameters is seen when comparing scenario S_all _with the unlabeled reference (Figure 8). The line is shifted about a factor of 10 to the left. The increase is even more pronounced for the most accurate estimation (0.92% that is about a factor of 50). With up to 70% standard deviation, 10 parameters are found for the labeled experiment compared to only two for the reference.

When comparing the three labeled scenarios only little differences can be found. With some additional labeling information (scenario S_C,D-frag_) the estimates become slightly more accurate (red, dash-dotted line). A missing concentration (scenario S_D-rel_) can obviously be compensated by the labeling information. The dashed line is shifted to the right slightly. The most accurate estimate has a standard deviation of 0.93%.

These findings are also reflected in the *D*- and *A*-criterion (Table 4). The *A*-criterion is based on the variances and reflects an information increase that is comparable to observations from Figure 8. The increase in information is 11.01 (scenario S_all_). The information gain from scenario S_C,D-frag _is about 1% higher; for S_D-rel _about 1% lower.

**Table 4:** Comparison of the D- and A-crtiteria.

		**Scenario**		
		
Substrate	Criterion	S_all_	S_D-rel_	S_C,D-frag_
^12^C, ***ξ***	*D*	2.20 10^8^		
	*I*_*D*_	1		
U-^13^C_2_, ***ξ***	*D*	5.05 10^-26^	2.57 10^-25^	3.58 10^-27^
	*I*_*D*_	5.02	4.85	5.31
^12^C, ***ξ***	*A*	1.06 10^6^		
	*I*_*A*_	1		
U-^13^C_2_, ***ξ***	*A*	1.02 10^3^	1.07 10^3^	0.91 10^3^
	*I*_*A*_	10.17	9.95	10.77

List of D- and A-criteria for the different measurement scenarios of an experiment without the addition of labeling (^12^C) and an experiment with fully labeled substrate (U-^13^C_2_). The calculation of D, A and the information index is given in Eq. (11) and (12).

The information gain calculated from the *D*-criterion is lower. Here a value of 6.91 is calculated for scenario S_all_. With some more available labeling measurements (scenario S_C,D-frag_) a factor of 7.31 is found. The information gain for scenario S_D-rel _decreases only marginally compared to S_all _(6.80).

### Parameter correlations

**Figure 9:**
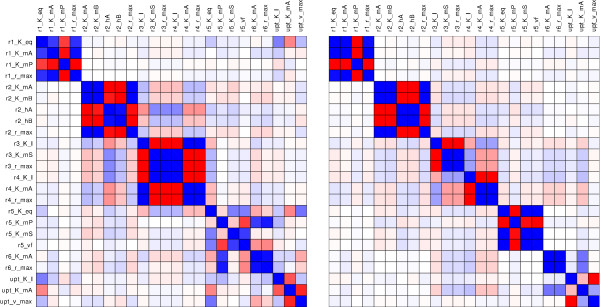
**Color visualization of the correlation matrix**. The parameter correlation matrix for the reference experiment (left) and the labeled experiment S_all _(right). Deep blue represents a correlation of 1, deep red represents -1.

So far, the improvement of the absolute variances of all parameters was investigated. In order to characterize the influence of the additional information from ^13^C labeling on the relationship among different parameters, the correlation matrix (Figure 9) will now be analyzed. The correlations for the reference experiment and the labeled scenario S_all _are shown. The parameters are grouped with respect to the reactions and a block structure becomes visible.

At first glance a strong decrease in correlation can be observed on the areas outside of the main diagonal blocks. There are blocks with only little changes close to the main diagonal. These blocks are formed from groups of parameters that belong to one single reaction step. For example, the kinetic parameters *v*_*max *_and *K*_*mA *_of the reaction v_1 _are strongly positive correlated (bue). I.e. if one is estimated too high, the other will also be estimated too high. Also in the labeled scenario, these parameters cannot be determined separately. It is well known from enzyme kinetic studies that *v*_*max *_and *K*_*mA *_values can hardly be independently determined, if the experimental concentration range is not chosen correctly.

In the following it will be seen that the effect of improved parameter distinction between different reaction steps is based on indirect flux measurements. The parameters *K*_*mA *_(of reaction v_1_) and *K*_*I *_(of v_upt_) were chosen here as an example, because a strong decrease in correlation is observed. In Figure 7 the sensitivities of these parameters are plotted for the reference and the scenario S_all_. Clearly, parameters with distinct influence on measurements will be less correlated. Within the reference the parameters exhibit a rather comparable influence on all measurements (though the magnitude is distinct). As a consequence, positive correlation is observed.

If labeling is introduced, the picture gets different. The influences on the measured mass isotopomers show some behavior that leads to a decoupling of parameters especially within the first 5 s after the pulse. The intermediate labeling patterns, particularly E+2, B+2 as well as E+0 and B+0 exhibit a distinct behavior for parameters *K*_*mA *_and *K*_*I*_. Whereas *K*_*mA *_exhibits a strong influence on the time course of these mass isotopomers, the influence of *K*_*I *_is relatively small. Additionally, the sensitivities of *K*_*mA *_show a strong time dependency that increases the independent measurement of this parameter.

## Conclusion

The exploratory study strongly suggests that performing a stimulus response experiment with ^13^C labeled substrate and measuring the intracellular labeling states can significantly increase the accuracy of kinetic parameter estimation. A decrease in the variance of more than a factor of 10 was observed for the analyzed example network (Figure 8). With labeling, three parameters are determined with less than 10% relative standard deviation. More than 40% of the parameters (i.e. 10 of 24) can be determined with less than 70% standard deviation (Figure 8).

Additionally it was found that various parameter correlations are drastically reduced (Figure 9). When comparing the experiment with labeled substrate to its reference counterpart a seven fold information gain (based on the *D*-criterion) was observed. Interestingly, the information loss due to the unknown concentration of pool D (scenario S_all_) can be compensated by the measured mass isotopomer distributions. This scenario shows only slightly less accurate determined parameters (Figure 8 and Table 4)

It can clearly be seen from the sensitivities that the parameters influence the labeling time course more specifically than the concentration time course. When only measuring the concentrations, a concentration increase can be explained by an increased influx or a decreased efflux. The addition of isotopic transients to the concentration courses serves as additional information about the pool exchange. A higher influx will lead to a faster labeling enrichment, while a decreased efflux will slow down the labeling enrichment. Thus, the labeling transients include information about the pool exchange that delivers valuable constraints for parameter identification.

In the case of isotopically nonstationary experiments under metabolic steady-state conditions, a similar example network was analyzed to estimate the information gain compared to a classical ^13^C labeling experiment at isotopically steady-state [39]. The conclusions from the simulation study have shown to be in accordance with findings from a complex *E. coli *network [11]. Therefore, the results obtained from the performed exploratory study should be transferable to real sized metabolic networks.

Clearly, an automated simulation tool is required to set up the equations based on the stoichiometry and the atom transitions. To solve these equations algorithms comparable to the tool of Nöh et al. [29] could be applied. Nöh et al. [29] have shown that the solution of approximately 4000 equations can be handled without problems. Compared to this number, the additional kinetic equations (1) can be neglected. Also, the sensitivities can be rapidly calculated using a cluster computer [29]. As further refinement, Antoniewicz et al. [28] demonstrated that the dimension of isotopomer balances can be further reduced using the concept of Elementary Metabolite Units (EMU).

## Authors' contributions

SAW: Did the simulations and statistical evaluation, writing of the manuscript, KN: Wrote the section about statistical measures, correction of the manuscript, WW: Supervision of the presented work, correction of the manuscript.

All authors read and approved the manuscript.

## Appendix: Details on the measurement model

Within scenario S_all _all pool concentrations can be measured and the labeling state is observed by mass isotopomers from unfragmented molecules over time (Section "Scenarios"). For a metabolite with *n *carbon atoms, a total of *n*+1 different mass isotopomers will be measured. For example, the unlabeled pool A#00 will be found at a mass M(A)+0 (henceforth denoted as A+0). The signal A+1 is evoked by the singly labeled isotopomers A#01 and A#10. The fully labeled A#11 is found in the signal A+2. Usually some linear relationship between the signal intensity *y*_A _and the concentration can be supposed. A scalable measurement model with some scaling factor ω is introduced by Möllney et al. [10]. Following this approach, the mass isotopomer measurements for pool A can be expressed by:

*y*_+0_(*t*_*k*_) = ω(*t*_*k*_)·*a*_00_(*t*_*k*_)

*y*_+1_(*t*_*k*_) = ω(*t*_*k*_)·(*a*_01_(*t*_*k*_) + *a*_10_(*t*_*k*_))

*y*_+2_(*t*_*k*_) = ω(*t*_*k*_)·*a*_11_(*t*_*k*_)

which can be shortly denoted by

(16) **y**(*t*_*k*_) = ω(*t*_*k*_)·**M x**(*t*_*k*_)

The measurement equations with tandem mass isotopomers [37] are a little bit more evolved. The construction of the associated measurement matrix can be found in [37]. Its implementation is then done analogously to the example shown.

In contrast to an experiment without labeling, a metabolite pool is now distributed over various mass isotopomers depending on the labeling state. The total concentration has to be reconstructed from the single mass isotopomer measurements. Assuming that the labeling has no influence on the ionization, the calibration for the unlabeled metabolite can be used for all mass traces. Usually some linear relationship between the signal intensity *y *and the sample concentration is supposed, given by the response factor γ. The sample concentration originates from various pre-processing steps (Figure 6). Thus, the measured signal is obtained from the intracellular concentration *c*(*t*_*k*_), the dilution factor φ(*t*_*k*_) (sample preparation) and the response factor γ:

(17) $$\begin{array}{ccc} y\left(t_{k}\right)=\gamma \varphi \left(t_{k}\right)\;c(t_{k}) & \text{with} & c(t_{k})=\sum_{i=0}^{n}c_{+i}\left(t_{k}\right) \end{array}$$

Some specialities of the different factors have to be pointed out:

1. The response factor γ is a device dependent factor that will usually be constant for all measurement time points, but different for each metabolite:

(18) γ = (γ_A_,...,γ_E_)^*T*^

2. The dilution factor φ(*t*_*k*_) reflects the sampling procedure and, thus, is time dependent (e.g. due to different amounts for neutralization). With regard to the metabolites, different scenarios could be taken into account. Current observations show, that during quenching metabolites leak to different extents [40]. Therefore in this study, a dilution factor for each metabolite is introduced that is independent. The vector of dilution factors thus reads:

(19) **φ**(**ξ**) = (φ_A_(*t*_1_),...,φ_E_(*t*_1_),...,φ_A_(*t*_*N*_),...,φ_E_(*t*_*N*_))^*T*^

Here a difference between the labeled and the non-labeled experiment has to be noticed. The dilution factor φ(*t*_*k*_) is a pool factor, the isotopomers of this pool are diluted to the same extent.

3. The scaling factor **ω**(*t*_*k*_) is used for scaling the simulated labeling enrichments to the absolute scale of the measurement device. Consequently it can be replaced by the previously discussed scaling factors:

(20) **ω**(**ξ**) = (**γ**_A _φ_A_(*t*_1_)/*A*, **γ**_B _φ_B_(*t*_1_)/*B*,..., **γ**_E _φ_E_(*t*_*N*_)/*E*)^*T*^

The errors of the single factors used for the simulation study have been mentioned in the main text. How these errors can be included to the regression model has not been discussed. The response factor γ is determined from a standard addition and is assumed to be constant over the measurement sequence. This factor is determined for each metabolite.

In contrast, φ(*t*_*k*_) is needed for each measurement time point. Thus, the regression Eq. (8) has to be extended by the scaling factors γ and φ(*t*_*k*_):

(21) $$\left(\begin{array}{c} \eta \left(\xi \right)\\ \vdots \\ {\tilde{\varphi }}_{\text{A}}({\text{t}}_{1})\\ {\tilde{\varphi }}_{\text{B}}({\text{t}}_{1})\\ \vdots \\ {\tilde{\varphi }}_{\text{E}}({\text{t}}_{\text{N}})\\ {\tilde{\gamma }}_{\text{A}}\\ \vdots \\ {\tilde{\gamma }}_{\text{E}} \end{array}\right)=\left(\begin{array}{c} g\left(x\left(\xi \right),c\left(\xi \right)\right)\\ \vdots \\ \varphi_{\text{A}}({\text{t}}_{1})\\ \varphi_{\text{B}}({\text{t}}_{2})\\ \vdots \\ \varphi_{\text{E}}({\text{t}}_{\text{N}})\\ \gamma_{\text{A}}\\ \vdots \\ \gamma_{\text{E}} \end{array}\right)+\left(\begin{array}{c} \varepsilon \left(\xi \right)\\ \vdots \\ \varepsilon_{\varphi_{\text{A}}}({\text{t}}_{1})\\ \varepsilon_{\varphi_{\text{B}}}({\text{t}}_{2})\\ \vdots \\ \varepsilon_{\varphi_{\text{E}}}({\text{t}}_{\text{N}})\\ \varepsilon_{\gamma \text{A}}\\ \vdots \\ \varepsilon_{\gamma \text{E}} \end{array}\right)$$

### Construction of the measurement covariance matrix

The covariance matrix **Σ **contains the measurement variances that can be obtained from multiple measurements or more common from measurement validation. Its construction depends on the regression model (Eq. (21)). In case that **g**(**x**(**ξ**), **c**(**ξ**)) calculates the measurement vector (A_+0_, A_+1_, A_+2_, B_+0_, B_+1_, B_+2_, C_+0_, C_+1_, ... E_+2_) at *t*_1 _and in the following of *t*_2 _until *t*_*N*_. For sample preparation we have to take into account the dilution (**Φ**) of each metabolite at each time point. The accuracy of the concentration-response is determined for each metabolite. Thus, the matrix will have following structure:

$$\begin{array}{l} \Sigma_{\text{MI}}=\left(\begin{array}{ccccccccc} \text{Var}({\text{A}}_{+0}({\text{t}}_{1})) & & & & & & & & \\ & \text{Var}({\text{A}}_{+1}({\text{t}}_{1})) & & & & & & & \\ & & \ddots & & & & & & \\ & & & \text{Var}({\text{E}}_{+2}({\text{t}}_{1})) & & & & & \\ & & & & \text{Var}({\text{A}}_{+0}({\text{t}}_{2})) & & & & \\ & & & & & \ddots & & & \\ & & & & & & \text{Var}({\text{E}}_{+2}({\text{t}}_{2})) & & \\ & & & & & & & \ddots & \\ & & & & & & & & \text{Var}({\text{E}}_{+2}({\text{t}}_{\text{N}})) \end{array}\right)\\ \Sigma_{\Phi }=\left(\begin{array}{ccccccccc} \text{Var}(\Phi_{\text{A}}({\text{t}}_{1})) & & & & & & & & \\ & \text{Var}(\Phi_{\text{B}}({\text{t}}_{1})) & & & & & & & \\ & & \ddots & & & & & & \\ & & & \text{Var}(\Phi_{\text{E}}({\text{t}}_{1})) & & & & & \\ & & & & \text{Var}(\Phi_{\text{A}}({\text{t}}_{2})) & & & & \\ & & & & & \ddots & & & \\ & & & & & & \text{Var}(\Phi_{\text{E}}({\text{t}}_{2})) & & \\ & & & & & & & \ddots & \\ & & & & & & & & \text{Var}(\Phi_{\text{E}}({\text{t}}_{\text{N}})) \end{array}\right)\\ \Sigma_{\gamma }=\left(\begin{array}{cccc} \text{Var}(\gamma_{\text{A}}) & & & \\ & \text{Var}(\gamma_{\text{B}}) & & \\ & & \ddots & \\ & & & \text{Var}(\gamma_{\text{E}}) \end{array}\right)\\ \Sigma_{\gamma }=\left(\begin{array}{ccc} \Sigma_{\text{MI}} & & \\ & \Sigma_{\Phi } & \\ & & \Sigma_{\gamma } \end{array}\right) \end{array}$$

## References

[B1] Kopka J (2006). Current challenges and developments in GC-MS based metabolite profiling technology. J Biotechnol.

[B2] Luo B, Grönke K, Takors R, Wandrey C, Oldiges M (2007). Simultaneous determination of multiple intracellular metabolites in glycolysis, pentose phosphate pathway and tricarboxylic acid cycle by liquid chromatography-mass spectrometry. J Chromatogr A.

[B3] Villas-Boas SG, Mas S, Akesson M, Smedsgaard J, Nielsen J (2005). Mass spectrometry in metabolome analysis. Mass Spectrom Rev.

[B4] Caponigro GP (1996). Mechanisms and control of mRNA turnover in Saccharomyces cerevisiae. Microbiol Rev.

[B5] Bendt AK, Burkovski A, Schäffer S, Bott M, Farwick M, Hermann T (2003). Towards a phosphoproteome map of Corynebacterium glutamicum. Proteomics.

[B6] Cornish-Bowden A, Cornish-Bowden A (1995). Fundamentals of Enzyme Kinetics.

[B7] Stephanopoulos G, Aristidou A, Nielsen J (1998). Metabolic Engineering: Principles and Methodogies.

[B8] Wiechert W, Möllney M, Petersen S, de Graaf AA (2001). A universal framework for C-13 metabolic flux analysis. Metab Eng.

[B9] Cohen SM, Glynn P, Shulman RG (1981). 13C NMR study of gluconeogenesis from labeled alanine in hepatocytes from euthyroid and hyperthyroid rats. Proc Natl Acad Sci U S A.

[B10] Möllney M, Wiechert W, Kownatzki D, de Graaf AA (1999). Bidirectional reaction steps in metabolic networks: IV. Optimal design of isotopomer labeling experiments. Biotechnol Bioeng.

[B11] Nöh K, Gronke K, Luo B, Takors R, Oldiges M, Wiechert W (2007). Metabolic flux analysis at ultra short time scale: isotopically non-stationary 13C labeling experiments. J Biotechnol.

[B12] Oldiges M, Takors R (2005). Applying metabolic profiling techniques for stimulus-response experiments: Chances and pitfalls. Adv Biochem Eng Biot.

[B13] Schäfer U, Boos W, Takors R, Weuster-Botz D (1999). Automated sampling device for monitoring intracellular metabolite dynamics. Anal Biochem.

[B14] Theobald UM (1997). In vivo analysis of metabolic dynamics in saccharomyces cerevisiae: I. Experimental observations. Biotechnol Bioeng.

[B15] Visser D, van Zuylen GA, van Dam JC, Oudshoorn A, Eman MR, Ras C, van Gulik WM, Frank J, van Dedem GWK, Heijnen JJ (2002). Rapid sampling for analysis of in vivo kinetics using the BioScope: A system for continuous-pulse experiments. Biotechnol Bioeng.

[B16] Buchholz A, Hurlebaus J, Wandrey C, Takors R (2002). Metabolomics: quantification of intracellular metabolite dynamics. Biomol Eng.

[B17] Buziol S, Bashir I, Baumeister A, Claaßen W, Noisommit-Rizzi N, Mailinger W, Reuss M (2002). New bioreactor-coupled rapid stopped-flow sampling technique for measurements of metabolite dynamics on a subsecond time scale. Biotechnol Bioeng.

[B18] Lange HC, Eman M, van Zuijlen G, Visser D, van Dam JC, Frank J, de Mattos MJT, Heijnen JJ (2001). Improved rapid sampling for in vivo kinetics of intracellular metabolites in Saccharomyces cerevisiae. Biotechnol Bioeng.

[B19] Oldiges M, Kunze M, Degenring D, Sprenger GA, Takors R (2004). Stimulation, monitoring, and analysis of pathway dynamics by metabolic profiling in the aromatic amino acid pathway. Biotechnol Progr.

[B20] Wahl SA, Haunschild MD, Oldiges M, Wiechert W (2006). Unravelling the regulatory structure of biochemical networks using stimulus response experiments and large-scale model selection. IEE Proc Syst Biol.

[B21] Selivanov VA, Puigjaner J, Sillero A, Centelles JJ, Ramos-Montoya A, Lee PW, Cascante M (2004). An optimized algorithm for flux estimation from isotopomer distribution in glucose metabolites. Bioinformatics.

[B22] Selivanov VA, Meshalkina LE, Solovjeva ON, Kuchel PW, Ramos-Montoya A, Kochetov GA, Lee PW, Cascante M (2005). Rapid simulation and analysis of isotopomer distributions using constraints based on enzyme mechanisms: an example from HT29 cancer cells. Bioinformatics.

[B23] Selivanov VA, Marin S, Lee PWN, Cascante M (2006). Software for dynamic analysis of tracer-based metabolomic data: estimation of metabolic fluxes and their statistical analysis. Bioinformatics.

[B24] Wiechert W, deGraaf AA (1997). Bidirectional Reaction Steps in Metabolic Networks I. Modeling and Simulation of Carbon Isotope Labeling Experiments. Biotechnol Bioeng.

[B25] Schuster S, Fell DA, Dandekar T (1999). A general definition of metabolic pathways useful for systematic organization and analysis of complex metabolic networks. Nat Biotechnol.

[B26] Wiechert W, Möllney M, Isermann N, Wurzel W, de Graaf AA (1999). Bidirectional reaction steps in metabolic networks: III. Explicit solution and analysis of isotopomer labeling systems. Biotechnol Bioeng.

[B27] van Winden WA, Heijnen JJ, Verheijen PJT (2002). Cumulative bondomers: A new concept in flux analysis from 2D [C-13,H-1] COSYNMR data. Biotechnol Bioeng.

[B28] Antoniewicz MR, Kelleher JK, Stephanopoulos G (2007). Elementary metabolite units (EMU): a novel framework for modeling isotopic distributions. Metab Eng.

[B29] Nöh K, Wahl A, Wiechert W (2006). Computational tools for isotopically instationary C-13 labeling experiments under metabolic steady state conditions. Metab Eng.

[B30] Wiechert W (2001). C-13 metabolic flux analysis. Metab Eng.

[B31] Bates DM, Watts DG (1988). Nonlinear Regression Analysis and its Applications.

[B32] Michalewicz ZF (2002). How to Solve It: Modern Heuristics.

[B33] Beyer HG (2001). The Theory of Evolution Strategies.

[B34] Deuflhard P, Bornemann F (2002). Scientific Computing with Ordinary Differential Equations. Texts in Applied Mathematics.

[B35] Joshi M, Seidel-Morgenstern A, Kremling A (2006). Exploiting the bootstrap method for quantifying parameter confidence intervals in dynamical systems. Metab Eng.

[B36] Chatterjee S, Hadi AS (1988). Sensitivity Analysis in Linear Regression, Probability and Mathematical Statistics.

[B37] Rantanen A, Rousu J, Kokkonen JT, Tarkiainen V, Ketola RA (2002). Computing positional isotopomer distributions from tandem mass spectrometric data. Metab Eng.

[B38] Bader A (1980). A systematic approach to standard addition methods in instrumental analysis. J Chem Edu.

[B39] Nöh K, Wiechert W (2006). Experimental design principles for isotopically instationary 13C labeling experiments. Biotechnol Bioeng.

[B40] Bolten CJ, Kiefer P, Letisse F, Portais JC, Wittmann C (2007). Sampling for metabolome analysis of microorganisms. Anal Chem.

